# A comparison of electrophysiological microrecording versus automatic MR-based segmentation to determine subthalamic nucleus boundaries

**DOI:** 10.1007/s00701-025-06619-z

**Published:** 2025-07-22

**Authors:** Camilla de Laurentis, Stéphane Thobois, Teodor Danaila, Chloe Laurencin, Gustavo Polo, Stéphane Prange, Emile Simon

**Affiliations:** 1 Functional Neurosurgery Unit, Hôpital Neurologique Pierre Wertheimer, Hospices Civils de Lyon, Bron, France; 2 Movement Disorders unit, Parkinson Expert Center, Hôpital Neurologique Pierre Wertheimer, Hospices Civils de Lyon, Bron, France; 3 Université Claude Bernard Lyon 1, Faculté de Médecine Lyon Sud Charles Mérieux, Oullins-Pierre-Bénite, France; 4 Lyon Neurosciences Research Center, CNRS UMR 5292 - INSERM UMRS 1028, Bron, France

**Keywords:** Deep brain stimulation, Electrophysiology, MRI-based segmentation, Parkinson’s disease, Ideal trajectory

## Abstract

**Purpose:**

Accurate placement of electrodes within the subthalamic nucleus is critical for deep brain stimulation (STN-DBS) in Parkinson’s disease (PD). Our objective was to compare microelectrode recording (MER) and an automatic MR-based segmentation tool (BrainLab Elements^TM^) for STN targeting and the determination of STN boundaries.

**Methods:**

Seventy-eight PD patients were included. Electrode placement within the STN and STN entry and exit points were determined by both methods and compared for concordance.

**Results:**

Of 344 trajectories, 269 were inside the STN, with good concordance of both techniques (Fleiss’ kappa 0.721, [95%CI 0.623, 0.819]). Concordance of MER and MR-based for the selection of the optimal trajectory was good (Fleiss’ kappa 0.693, [95%CI 0.578, 0.808]), with less than 2.75mm difference between MER and MR-based for the STN entry (upper limit of agreement 2.752 [95%CI 2.365 to 3.138] mm; lower limit of agreement -2.406 [95%CI -2.793 to -2.020] mm) and exit points (upper limit of agreement 2.750 [95%CI 2.351 to 3.149] mm; lower limit of agreement -2.577mm [95%CI -2.976 to -2.178]).

**Conclusion:**

We demonstrated that MER and MR-based segmentation have a good concordance to determine STN boundaries during DBS surgery.

## Introduction

Subthalamic nucleus (STN) deep brain stimulation (DBS) is one of the most efficient treatments for fluctuating patients with Parkinson’s disease (PD)[7, 14]. The clinical benefit depends on the optimal placement of the lead within the STN, notably its sensorimotor part [6, 11, 18]. Therefore, determining the boundaries of the STN is a major issue and may rely on different approaches [9, 12, 25].

The targeting of the STN can be performed using direct visualization of the nucleus by MRI, or indirect methods [26], more or less combined with electrophysiological recordings (MER) [9, 17, 25] and macrostimulation whereas surgery can be robot-assisted or frame-based [22]. Among these multiple approaches, MER is very precise in determining the entry point and exit point of the STN and can also be coupled with local field potential [1, 23, 28]. However MER may be time-consuming, need specific expertise and, according to some authors may increase the risk of intraoperative bleeding because of multiple trajectories to be tested [10].

Therefore, it is of great interest to compare the coordinates of the STN determined by MER versus far less invasive imaging approaches such as MRI. Such a comparison has revealed some discrepancies [2, 24]. However, the development of new and more and more precise perioperative imaging tools based on preoperative MRI and intraoperative CT may challenge the interest of MER in defining STN boundaries.

Among these, automatic MR-based segmentation can be performed using Elements^TM^, by Brainlab AG (Munich, Germany)[21]. This allows the creation of a subject-specific anatomical atlas via automatic recognition of anatomical structures with high consistency and accuracy [20], this being based on a patented synthetic tissue model and on MR datasets constantly updated. Together with the trajectory planning tool it may propose some trajectories to reach a specific target. The great value of this software is the ability to deform and adapt to each patient’s images, thus becoming patient-specific. However, it remains of major importance to confront these indirect imaging approaches to direct anatomical information such as the ones provided by electrophysiology, in order to determine the quality of this segmentation.

The objective of this study was to compare the intraoperative coordinates of the STN obtained by MER versus preoperative MR-based segmentation using Brainlab Elements^TM^.

## Methods

This monocentric prospective study includes consecutive PD patients, who underwent bilateral STN-DBS using a robotic arm (NeuroMate^TM^, Renishaw) at our Institution (Hôpital Neurologique Pierre Wertheimer, Lyon) between October 2018 and November 2023, following STROBE guidelines for reporting [27]. Sex, age at surgery, intraoperative adverse events, and results of the intraoperative macrostimulation were collected.

Before surgery, the neurosurgeon (ES) calculated on each side the stereotactic coordinates of the STN, using a combination of indirect (Talairach’s coordinates), direct targeting (visualizing the STN on 3D FLAIR MRI sequences) and atlas targeting (with a patient personalized atlas made with Brainlab Elements^TM^, Brainlab AG, Munich, Germany), for the central trajectory. Then, during surgery, 3 microelectrodes were used (central, anterolateral, and anteromedial trajectories), and their exact position was determined using a CBCT scan (Medtronic O-arm, Minneapolis) fused with the preoperative planning on MRI.

Three-channel MER recording was performed using the Alpha Omega NeuroSmart^TM^ console, with “STR-009-080-00” electrodes, and a NeuroFortis^TM^ microdrive (AlphaOmega, Mannheim, Germany).

For each of the 3 electrodes, the placement within or outside the STN as well as the depth of the entry and exit points of the nucleus along the electrode axis were determined by the neurosurgeon (ES) using MR-based reconstruction and by the neurologist (SP, ST, TD, or CL) using MER, blinded to each other. Based on their respective information, one electrode was selected as optimal independently by the neurologist and by the neurosurgeon. Then, macrosimulation was performed on the trajectory both agreed to consider the best.

Inter-rater reliability was compared for MER versus MR-based methods for the following outcomes: 1) the location of each electrode within or outside the STN and the choice of the optimal electrode using concordance analysis of MER versus MRS with Fleiss’ kappa [16]; 2) and the difference and limits of agreement of STN entry and exit points for the optimal electrode using Bland-Altman analysis change reference [15].

Standard descriptive statistics were used to compare binary variables with the Chi-square test or Z-score to calculate population proportions. A threshold of p < 0.05 was set for statistical significance, and 95% confidence intervals (95%CI) were determined, using R for statistical analysis. The study was approved by the local ethics committee (23-5439).

## Results

We enrolled 78 PD patients (25 females, 53 males), aged 37-70 years old (mean 59.3, median 61.5 years old) and with a disease duration of 4 to 21 years (mean 10.6, median 10). All patients showed preoperative levodopa sensitivity >50%, absence of significant cognitive impairment, and no surgical or general contraindication.

A total of 344 intraoperative trajectories were analyzed, resulting in 156 definitive electrodes implanted. Among them, 269 were inside the STN (78.2%, p =.017) and 47 were outside the STN according to both MER and Elements^TM^ (13.7%, p <.0001); 21 were positioned within the STN for the MER but not for Elements^TM^ (6.1%, p <.0001); and 7 were considered inside the STN using Elements^TM^, but not for MER (2%, p <.0001) (Table 1). Overall, the concordance of MER versus MR-based segmentation methods was good (Fleiss’ kappa 0.721 [95%CI 0.623, 0.819]).

**Table 1:**
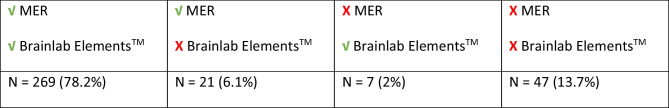
Trajectories inside/outside the subthalamic nucleus, sorted by technique

*MER* micro-electrophysiological recording

Regarding the trajectory considered optimal as determined independently by the neurologist using MER and neurosurgeon using MR-based segmentation only, the concordance was good (Fleiss’ kappa 0.693 [95%CI 0.578, 0.808]) (n = 156). Indeed, both approaches led to the same choice of trajectory in 133/156 cases (85.3%, p <.0001), whereas discrepancies were noted for 23 out of 156 electrodes (14.7%, p <.0001).

**Fig. 1:**
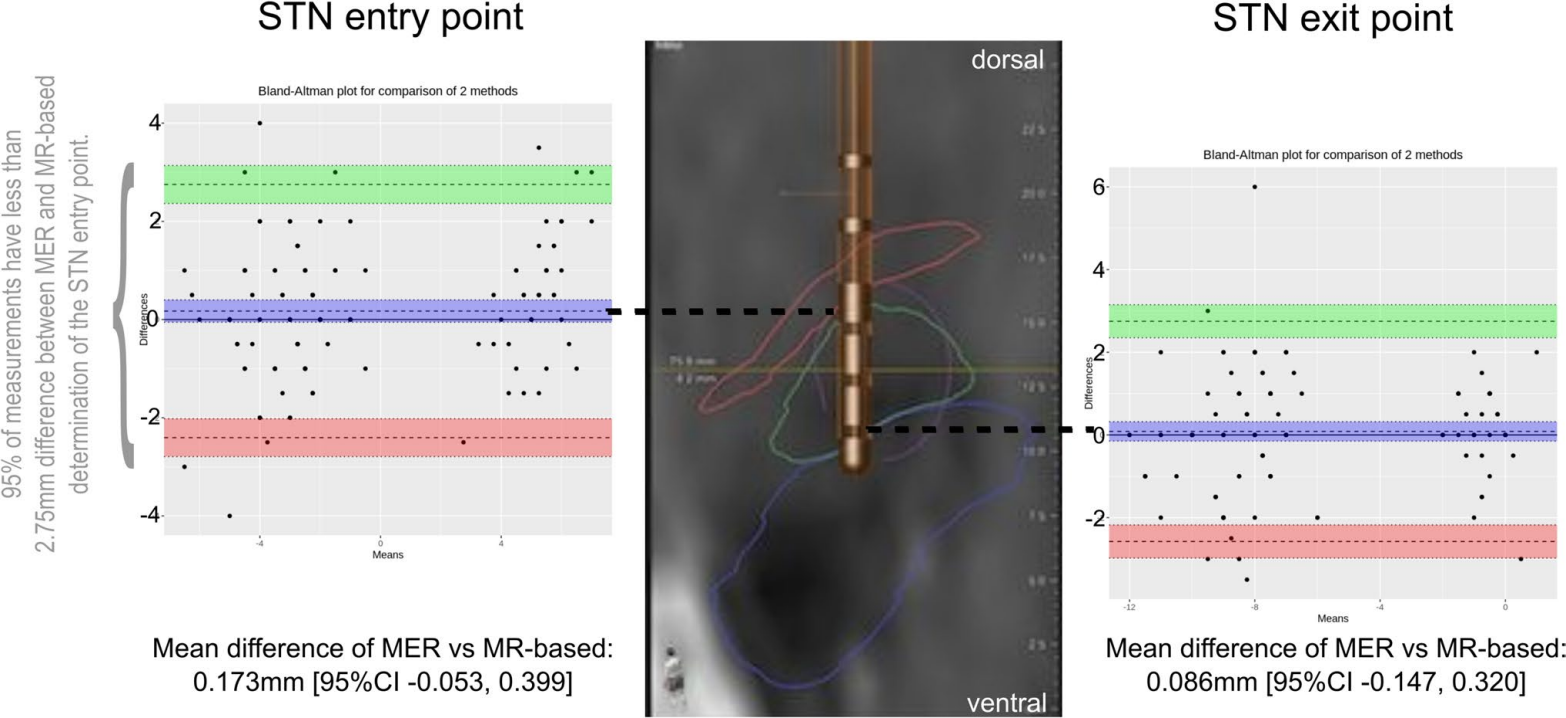
Schematic representation of the subthalamic nucleus boundaries (green) showing the Bland-Altman plots for the comparison of the difference of measurements between MER and MR-based segmentation for the entry (left) and exit (right) points of the subthalamic nucleus. For the entry point (left), a bias of 0.173 mm (95%CI −0.053 to 0.399) was calculated, with an upper limit of agreement (green) of 2.752mm (95%CI 2.365 to 3.138) and a lower limit of agreement (red) of −2.406 mm (95%CI −2.793 to −2.020). For the exit point (right), a bias of 0.086 mm (95%CI −0.147 to 0.320) was calculated, with an upper limit of agreement of 2.750mm (95%CI 2.351 to 3.149) and a lower limit of agreement of −2.577mm (95%CI −2.976 to −2.178)

The comparison of coordinates revealed a low mean difference between the two methods for the entry and exit points as determined by the two methods, with 0.173 mm [95%CI −0.053, 0.399] for the entry point and 0.086 mm [95%CI −0.147, 0.320] for the exit point (Fig. 1). Upper and lower limits of agreement are graphically displayed in Fig. 1, indicating that the difference was inferior to 2.75mm for 95% of the measurements between the MER and MR-based STN entry (upper limit of agreement 2.752 [95%CI 2.365 to 3.138] mm; lower limit of agreement −2.406 [95%CI −2.793 to −2.020] mm) and exit points (upper limit of agreement 2.750 [95%CI 2.351 to 3.149] mm; lower limit of agreement −2.577mm [95%CI −2.976 to −2.178]). A difference inferior to 2 mm for the entry and/or exit point was found in 119/133 (89.5%).

Before definitive implantation, we performed macrostimulation for 132/133 electrodes, resulting in a trajectory change for 13 electrodes (9.8%), to avoid side effects at low stimulation threshold. Eventually, concordance was similar for definitive electrodes using all three methods (MER versus macrostimulation: Fleiss’ kappa 0.645 [95%CI 0.524,0.766]; MR-based segmentation versus macrostimulation: Fleiss’ kappa 0.671 [95%CI 0.558,0.783]).

## Discussion

The present study demonstrates the good concordance of MER and MR-based automatic segmentation using Elements^TM^ to determine the boundaries of the STN and for the placement of electrodes during DBS surgery.

In previous studies, in which the electrode placement using either MER or MRI sequences or atlases were compared, some discrepancies were noted [2, 5, 13, 19, 24, 29].

On the contrary, our findings demonstrate the excellent concordance of this more recent automated imaging approach with MER to target the STN and, in turn, precisely place the electrodes.

We found that the coordinates of STN entry and exit points using Elements^TM^ and MER are similar, with 95% of the measurements within ±2.75mm, in line with previous findings [21]. We observed that the discrepancy was slightly greater for the exit point, possibly indicating lower precision using MER to distinguish the STN from the substantia nigra electrophysiological pattern [1, 3].

These results suggest that accurate placement of electrodes may be achieved using MR-based methods only. It is worth mentioning that some surgical teams have been successfully using direct targeting for a long time now, e.g. with a single trajectory and without MER [4, 8].

**Fig. 2:**
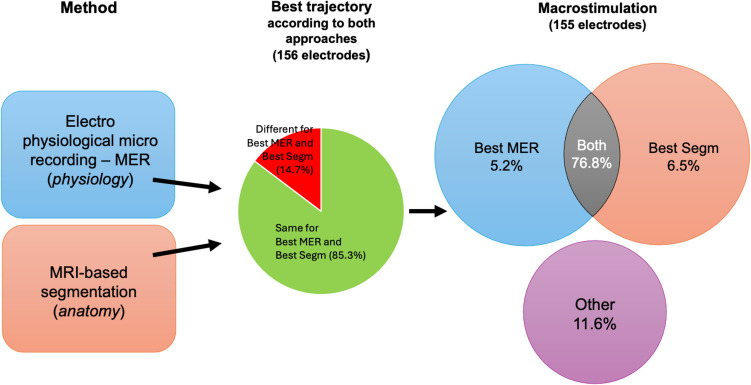
Flowchart synthesizing the workflow illustrated in the present paper. Both methods, electro physiological microrecording (MER) and MRI-based segmentation, were used to determine the best trajectory for each electrode (left). The two methods agreed on choosing the same best trajectory in 85.3% of cases (center). Macrostimulation was performed to select the definitive trajectory for the electrode to be implanted (right): in 76.8% of cases, it confirmed the best trajectory already suggested by both methods; in 5.2% it agreed with the best trajectory of MER, and in 6.5% with the best trajectory of MRI-segmentation. In 11.6% of the studied trajectories, macrostimulation suggested a trajectory other than the best ones of MER and MRI-segmentation as the best to be chosen for implantation

Besides the comparison between MER and automated imaging approach, the present series underlines that macrostimulation remains important as it led, in about 10% of the cases, to a trajectory change. In this context, Fig. 2 graphically shows our workflow.

## Conclusion

The present study suggests that preoperative MR-based segmentation of the STN combined with intraoperative stereotactic CT offers a similar accuracy for the placement of electrodes within the STN in comparison to intraoperative microrecording. As such, MR-based segmentation and electrode placement using ElementsTM methods could provide greater confidence to perform STN-DBS under general anesthesia without MER, reducing the number of trajectories before definitive implantation and, in turn, limiting the risk of bleeding, improving patients’ comfort and reducing procedures duration. This opens the way to randomized clinical trials to investigate the long-term clinical outcomes of DBS surgery using either surgical technique.

## Data Availability

No datasets were generated or analysed during the current study.

## Abbreviations

CBCT: cone beam computer tomography
DBS: deep brain stimulation
MER: micro-electrophysiological recordings
MR: magnetic resonance
MRI: magnetic resonance imaging
PD: Parkinson’s disease
STN: subthalamic nucleus
STROBE: The Strengthening the Reporting of Observational Studies in Epidemiology guidelines

## Declarations

### Study approval statement

This study protocol was reviewed and approved by the local ethics committee of the Hospices Civils de Lyon (Comité Scientifique et Éthique des Hospices Civils de Lyon), IRB (Internal Review Board) 00013204, AGORA number 23-5439.
